# Different inter­molecular inter­actions in solvated and unsolvated isatin-based di­thio­carbazate imine derivatives

**DOI:** 10.1107/S2056989025011028

**Published:** 2026-01-01

**Authors:** Aidan P. McKay, David B. Cordes, Mohd Abdul Fatah Abdul Manan

**Affiliations:** a EaStCHEM School of Chemistry, University of St Andrews, St Andrews, Fife KY16 9ST, United Kingdom; b Faculty of Applied Sciences, Universiti Teknologi MARA, 40450 Shah Alam, Selangor, Malaysia; University of Aberdeen, United Kingdom

**Keywords:** crystal structure, isatin, hydrogen bonding, chalcogen bonding

## Abstract

The syntheses and structures of 2-fluoro­benzyl (*Z*)-2-(2-oxoindolin-3-yl­idene)hydrazine-1-carbodi­thio­ate dimethyl sulfoxide monosolvate, C_16_H_12_FN_3_OS_2_·C_2_H_6_OS (**1**) and 2-fluoro­benzyl (*Z*)-2-(5-bromo-2-oxoindolin-3-yl­idene)hydrazine-1-carbodi­thio­ate, C_16_H_11_BrFN_3_OS_2_ (**2**) are reported. Both structures feature a *Z*-configuration with respect to the C=N bond and the fluoro­benzyl ring is approximately orthogonal to the isatin moiety. For **1**, the crystal packing features weak C_ar_—H⋯S (ar = aromatic) hydrogen bonds that link adjacent mol­ecules in a *C*(10) fashion to form pleated chains propagating along [001] and short S⋯O contacts between dimethyl sulfoxide solvent mol­ecules forming chains along [010]. The N—H hydrogen bond donors in **1** form either intra­molecular or discrete N—H⋯O_(DMSO)_ hydrogen bonds. In **2**, alternating *R*^2^_2_(8)-type pairwise N—H⋯O hydrogen bonds and short F⋯Br contacts link the mol­ecules into chains propagating along [210]. The later unsolvated structure is of notably poorer quality and exhibits disorder in its *o*-fluoro­benzyl group, with a 180° flip and a small twist around the S—C bond. These findings are consistent with the results of Hirshfeld surface analyses.

## Abstract 2

The crystal structures of a solvated and an unsolvated di­thio­carbazate imine derivatives are compared and contrasted.

## 1. Chemical context

The use of solvent mol­ecules in reducing structural disorder is an emerging theme in crystal engineering. Controlled solvation can enhance crystallographic quality and provides a means to fine-tune the chemical and physical properties of solid-state materials, including functional organic frameworks, coordination complexes and pharmaceuticals (for recent reviews, see: Werner & Swift, 2021 ▸; Bolla *et al.*, 2022 ▸). The inclusion of a solvent such as dimethyl sulfoxide (DMSO), in the crystal fills void space, introduces additional hydrogen-bond acceptors, and stabilizes conformations, leading to more rigid and well-defined structural units of crystalline motifs (Klitou *et al.*, 2020 ▸; Li *et al.*, 2021 ▸). For example, our recent crystallographic study on 2-fluoro­benzyl (*Z*)-2-(5-chloro-2-oxoindolin-3-yl­idene) hydrazine-1-carbodi­thio­ate dimethyl sulfoxide monosolvate highlighted the role of DMSO as a hydrogen bond acceptor by forming discrete directional N—H⋯O_(DMSO)_ contacts and occupying voids in the crystal. The inclusion of the solvate increased packing complementarity, producing a more rigid asymmetric unit and fewer alternative conformers, resulting in a more stable mol­ecular conformation (McKay *et al.*, 2025 ▸).

Isatin-based imines have been widely recognized as versatile scaffolds, displaying a broad range of application domains (Liu *et al.*, 2025 ▸; Tok *et al.*, 2025 ▸; Topkaya *et al.*, 2024 ▸). Their functionalization allows chemists to modulate steric and electronic properties for tuning solid-state architectures, which can exhibit profound implications on physical properties and applications (Wang *et al.*, 2023 ▸; Venugopal & Pansare 2025 ▸).

As part of our ongoing studies of organosulfur imines and their solid-state behaviour, we report here the syntheses, crystal structures and Hirshfeld-surface analyses of 2-fluoro­benzyl (*Z*)-2-(2-oxoindolin-3-yl­idene)hydrazine-1-carbodi­thio­ate dimethyl sulfoxide monosolvate, C_16_H_12_FN_3_OS_2_·C_2_H_6_OS (**1**) and 2-fluoro­benzyl (*Z*)-2-(5-bromo-2-oxoindolin-3-yl­idene)hydrazine-1-carbodi­thio­ate, C_16_H_11_BrFN_3_OS_2_ (**2**).
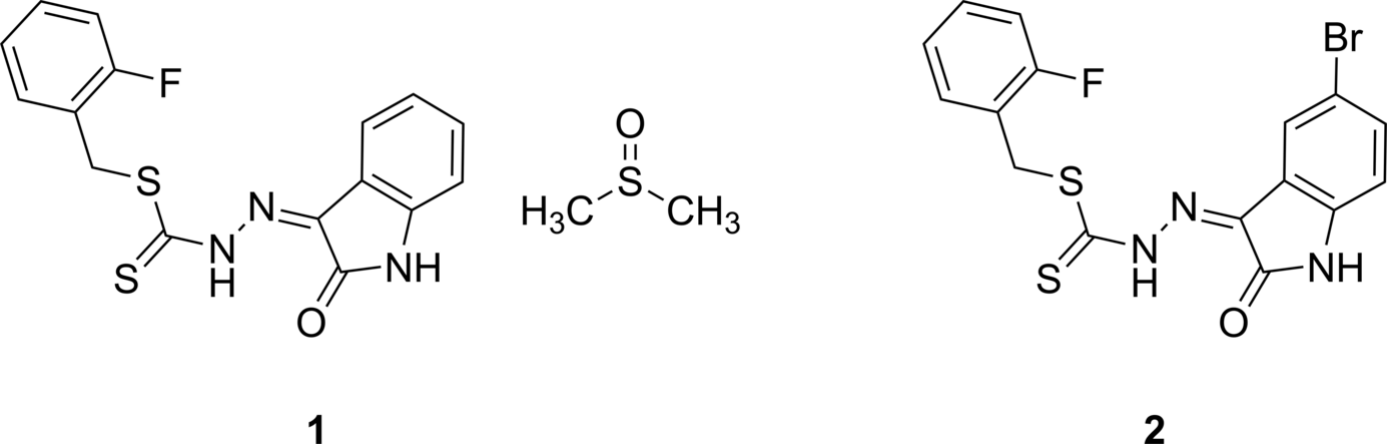


## 2. Structural commentary

**Figure 1:**
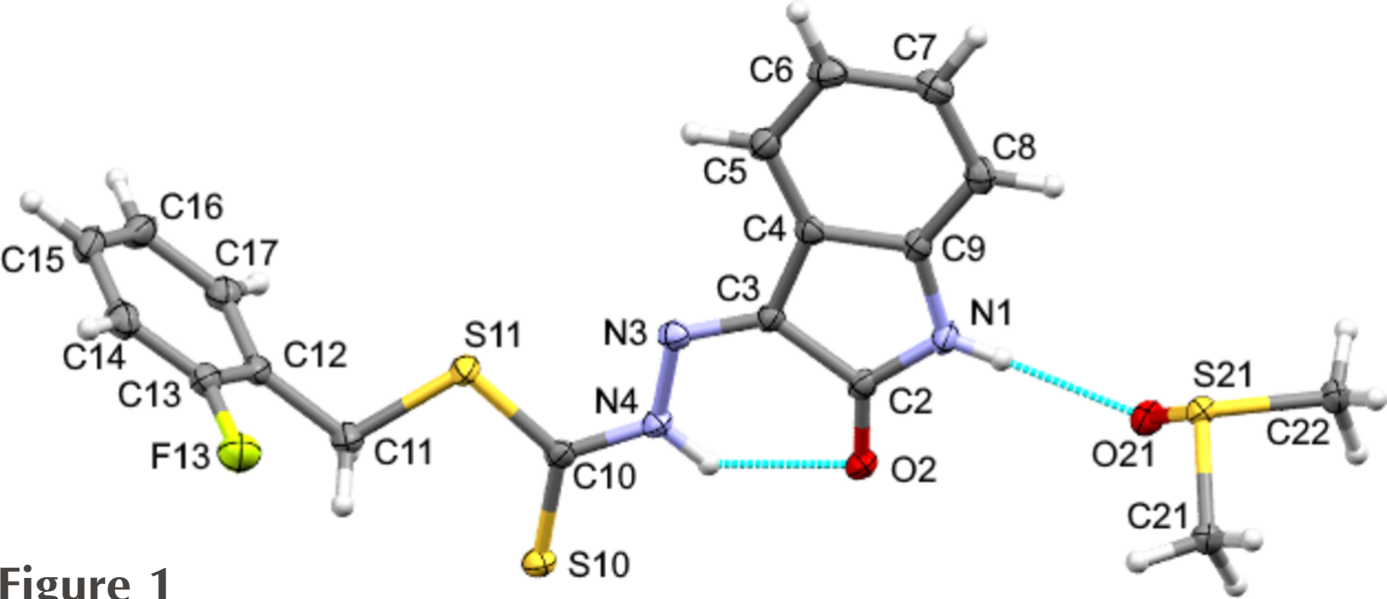
The mol­ecular structure of **1** with ellipsoids drawn at the 50% probability level. Hydrogen bonds are shown as blue dashed lines.

**Figure 2:**
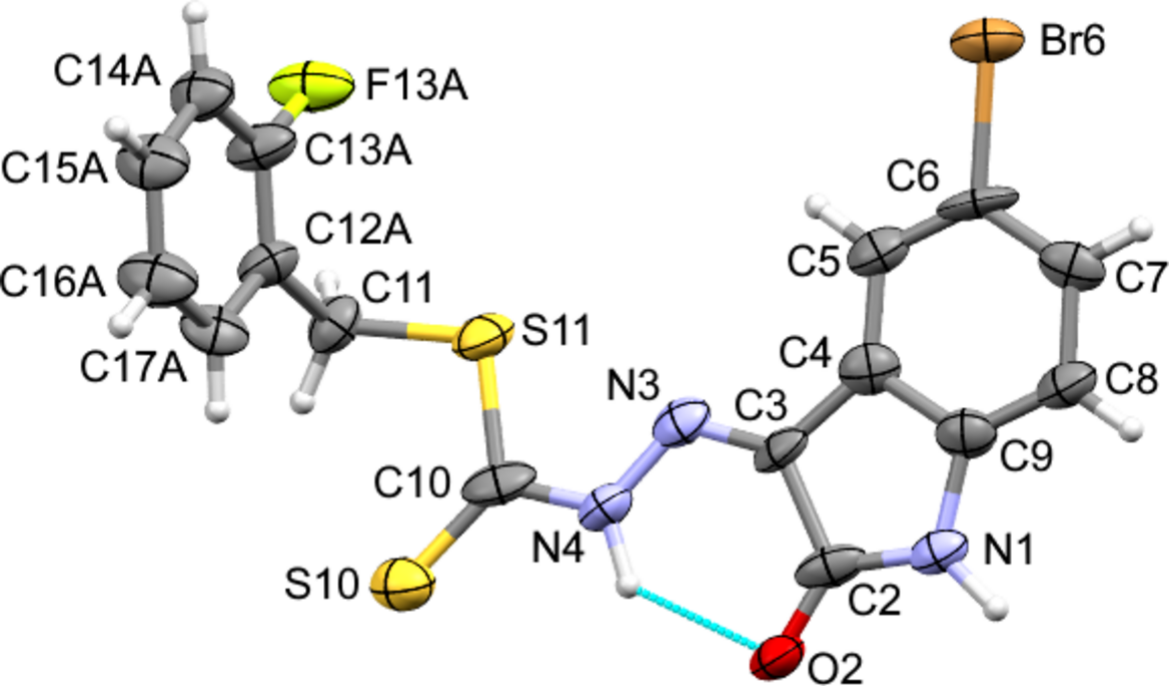
The mol­ecular structure of **2** with ellipsoids drawn at 50% probability and the minor component of disorder omitted for clarity. Hydrogen bonds are shown as blue dashed lines.

Compound **1** crystallizes in space group *P*2_1_/*c* as a dimethyl sulfoxide solvate (Fig. 1 ▸), while compound **2** crystallizes in the space group *P*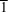, without any solvent of crystallization (Fig. 2 ▸). Both compounds display a *Z* configuration with respect to the C=N bond, resulting in the hydrazine (N4) hydrogen atom being directed towards the isatin (O2) oxygen atom generating an *S*(6) intra­molecular N—H⋯O hydrogen bond (Tables 1 ▸ and 2 ▸) in the same manner as in previously reported related structures (Abdul Manan *et al.*, 2023 ▸; Abdul Manan *et al.*, 2024*a* ▸,*b* ▸,*c* ▸; McKay *et al.*, 2025 ▸). In both structures, the thione sulfur atom (S10) is orientated *syn* to the γ-lactam oxygen atom while the S-2-fluoro­benzyl moiety is orientated in the opposite direction. There is a small bow between the methyl­idenehydrazinecarbodi­thio­ate (MHT) and the γ-lactam moieties in both structures [6.70 (8) and 6.5 (3)° for **1** and **2**, respectively] while the 2-fluoro­phenyl ring is twisted almost perpendicular to the MHT fragment [90.37 (7) and 72.5 (17)° for **1** and **2**, respectively]. In compound **1**, the H atom on the γ-lactam nitro­gen atom (N1) forms a discrete N—H⋯O hydrogen bond to the DMSO solvent mol­ecule (Table 1 ▸) in the same manner as the previously reported 5-chloro analogue (McKay *et al.*, 2025 ▸).

## 3. Supra­molecular features

**Figure 3:**
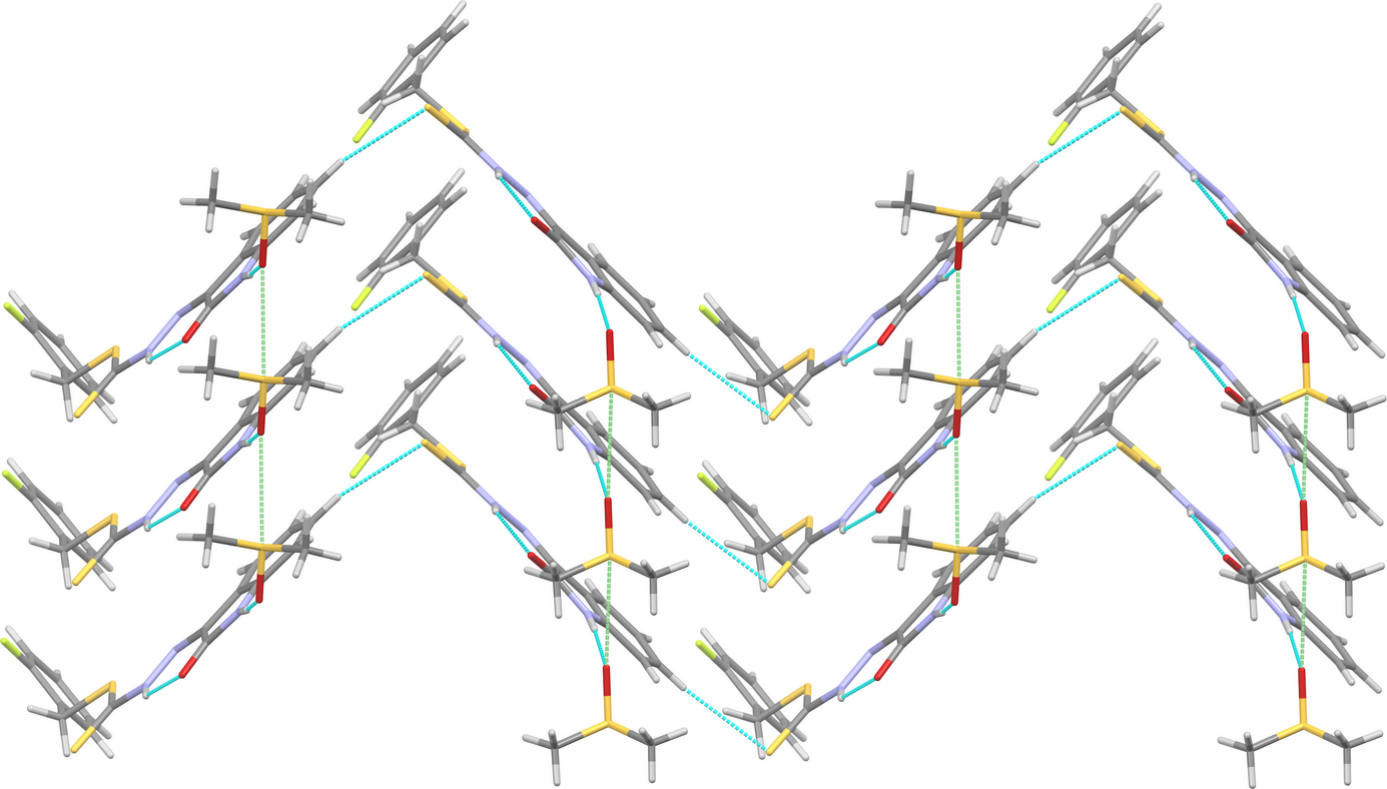
The packing of **1** into pleated sheets (left to right) through classical and non-classical hydrogen bonds (blue dashed lines) with chalcogen bonded (green dashed lines) DMSO solvent mol­ecules.

In the extended structure of **1** the fused benzo-ring of the isatin moiety forms non-classical C_ar_—H⋯S and C_ar_—H⋯F hydrogen bonds to the thione and the fluorine atoms (Table 1 ▸). These inter­actions link adjacent mol­ecules in a *C*(10) fashion (with respect to the C—H⋯S hydrogen bond) to form pleated chains propagating along [001] (Fig. 3 ▸). These pleated chains are similar to the pleated sheets reported in methyl (*Z*)-methyl 2-(−5-methyl-2-oxoindolin-3-yl­idene)hydrazine-1-carbodi­thio­ate (Abdul Manan *et al.*, 2024*c* ▸), which are formed by non-classical C_ar_—H⋯S hydrogen bonds between adjacent N—H⋯O hydrogen bonded dimers. The pleated chains in **1** further stack together through both weak π–π stacking (Corne *et al.*, 2016 ▸) from the fused benzo (C4–C9) of the isatin moiety to the C3=N3 bond [centroid⋯centroid separation = 3.302 (3) Å], and additional weaker non-classical C_ar_—H⋯S hydrogen bonds from C16 in the fluoro­benzyl ring to the sulfide group (S11) of an adjacent mol­ecule [H16⋯S11 = 3.0363 (6) Å, C16⋯S11 = 3.770 (3) Å]. This packing motif is supported by chains of chalcogen-bonded DMSO solvent mol­ecules [S21⋯O21 = 3.188 (2) Å compared to a van der Waals separation of 3.32 Å; S21—O21⋯S21 = 169.37 (10)°] along [010], which are hydrogen-bonded to the γ-lactam nitro­gen atoms to form sheets lying in the (100) plane.

**Figure 4:**
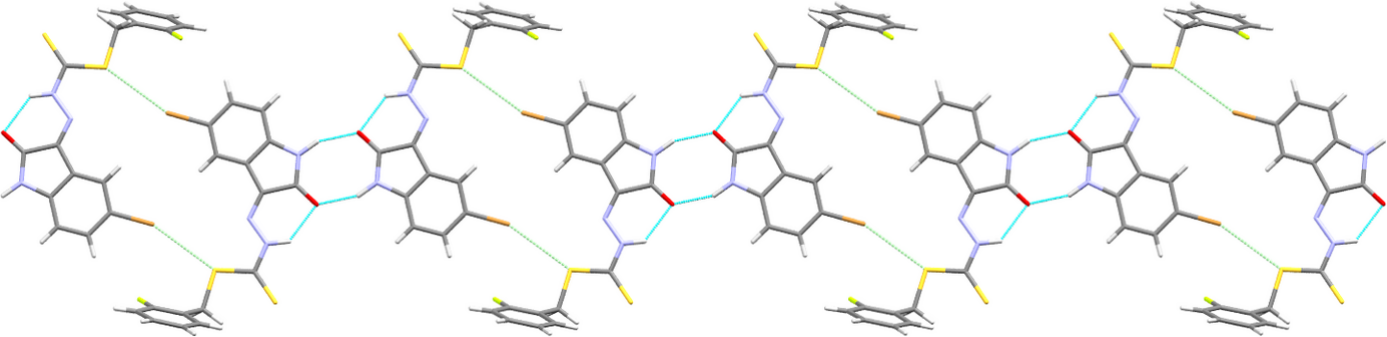
The packing of **2** into chains through alternating pairwise N—H⋯O hydrogen bonds (blue dashed lines) and S⋯Br halogen bonds (green dashed lines).

In contrast, **2** forms inversion dimers through reciprocal N—H⋯O hydrogen bonds in the typical 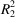
(8) form (Table 2 ▸) between adjacent γ-lactam units in the same manner as previously reported related structures (Abdul Manan *et al.*, 2023 ▸, 2024*a* ▸,*b* ▸,*c* ▸). The dimers link together into chains (Fig. 4 ▸) propagating along [2
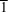
0] through Br⋯S halogen bonds (Br6⋯S11 = 3.405 (5) Å, C6—Br6⋯S11 = 158.7 (6)°). These chains pack together into sheets in (001) through a combination of edge-to-face stacking between the fused benzo rings (C8) and fluoro­phenyl rings [H8⋯centroid = 3.20 (2) and 3.22 (2) Å, C8⋯centroid 3.91 (3) and 3.98 (3) Å, for the major and minor disordered parts, respectively], π–π stacking between adjacent γ-lactam rings [centroid–centroid separation = 3.467 (13) Å], weaker non-classical C_ar_—H⋯S [H⋯S = 2.956 (4)–3.211 (4) Å, C⋯S = 3.68 (3)–4.01 (4) Å] and C_ar_—H⋯Br [H5⋯Br6 = 3.201 (2) Å; C5⋯Br6 = 4.138 (16) Å] hydrogen bonds, and, depending on the orientation of the *o*-fluoro­benzyl ring, either very weak Type IV (Saha *et al.*, 2023 ▸) Br⋯F [Br6⋯F13*A* = 3.35 (3) Å compared to a van der Waals separation of 3.37 Å; C6—Br6⋯F13*A* = 96.0 (7)°; C13A—F13*A*⋯Br6 = 104 (2)°] halogen bonds or weak non-classical C_ar_—H⋯F [H17*B*⋯F13*B* = 2.65 (2) Å; C17*B*⋯F13*B* = 3.26 (4) Å] hydrogen bonds. These sheets assemble into the overall structure through additional weaker C_ar_—H⋯S and C_ar_—H⋯F inter­actions.

## 4. Hirshfeld surface analysis

**Figure 5:**
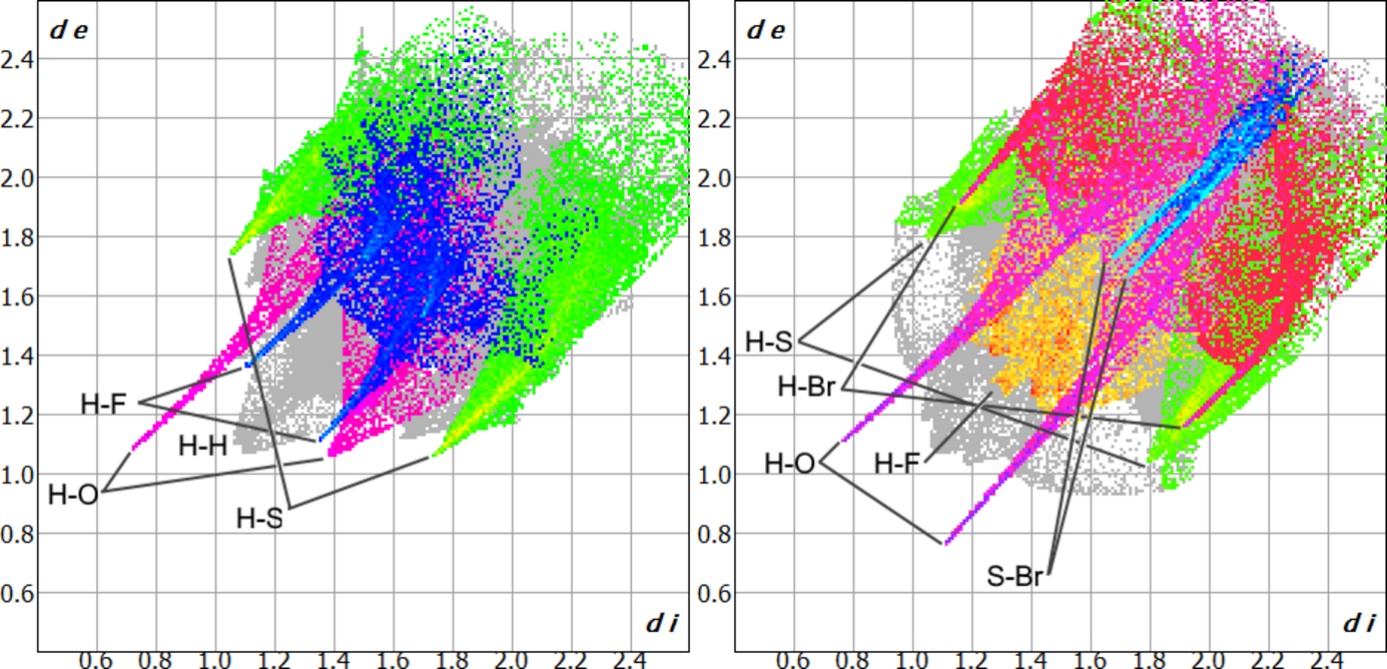
Hirshfeld surface fingerprint plots of **1** (left) and **2** (right) with various contacts highlighted.

Hirshfeld surface analyses of **1** (with the DMSO solvent mol­ecule external to the surface) and **2** (Fig. 5 ▸) both show sharp H⋯O and H⋯S contacts (5.9 and 7.5%, and 17.0 and 13.8% of the surfaces, respectively). The H⋯S fingerprints for both structures show broad tails, highlighting the diverse range of H⋯S contacts occurring beyond the discrete C—H⋯S non-classical hydrogen bonds mentioned above. The majority of these contacts are likely electrostatic/van der Waals in character. The fingerprint of H⋯Br contacts in **2** (8.3% of surface) shows a sharp contact which corresponds to a long C_ar_—H⋯Br inter­action [H5⋯Br6 = 3.201 (2) Å] supporting the halogen-bonding inter­action. The surface of **1** shows sharp H⋯F contacts (7.5% of surface) which correspond to a weak C_ar_—H⋯F inter­action [H6⋯F13 = 2.6116 (15) Å; C6⋯F13 = 3.540 (3) Å] between the fused benzo ring of the istan grouping and an adjacent fluoro­benzyl ring while the surface of **2** only shows diffuse H⋯F contacts (4.5% of surface), likely due to the disorder of the fluoro­benzyl group. The surface of **2** also shows sharp S⋯Br contacts (2.9% of surface) consistent with the halogen bonds observed in the structure.

## 5. Synthesis and crystallization

The 2-fluoro­benzyl hydrazinecarbodi­thio­ate precursor was synthesized using our previously published methods (McKay *et al.*, 2025 ▸).

To prepare **1**, a solution of isatin (1.47 g, 10.0 mmol, 1 eq.) in hot ethanol (40 ml) was added to a solution of the di­thio­carbazate precursor (2.16 g, 10.0 mmol, 1.0 e.q) in hot ethanol (40 ml). The mixture was heated (353 K) with continuous stirring for 15 min and later allowed to cool to room temperature and stand for about 20 min., until a precipitate formed, which was then collected by filtration and dried over silica gel. The crude solid was purified by recrystallization from ethanol solution to yield a yellow solid (yield: 2.87 g, 83%). m.p. 494–495 K; elemental analysis calculated for C_16_H_12_FN_3_OS_2_: C, 55.63; H, 3.50; N, 12.17%; found: C, 55.63; H, 3.12; N, 11.96%. FT–IR (KBr, *ν*, cm^−1^): 3175 (NH), 1688 (C=O); 1613 (C=N); 1079 (C=S); 1143 (N—N); ^1^H NMR (400 MHz, *d_6_*-DMSO) δ: (ppm): 4.56 (*s*, 2H), 6.94 (*d*, *J* = 7.9 Hz, 1H) 7.04–7.08 (*m*, 1H), 7.17–7.26 (*m*, 2H), 7.35–7.42 (*m*, 2H), 7.53–7.58 (*m*, 2H), 11.38 (*s*, 1H), 13.97 (*s*, 1H). Crystals of the DMSO solvate suitable for X-ray diffraction were grown by slow evaporation of a dimethyl sulfoxide solution at room temperature.

Compound **2** was prepared as follows: a solution of 5-bromo­isatin (2.26 g, 10.0 mmol, 1.0 eq.) in hot ethanol (40 ml) was added to a solution of the di­thio­carbazate precursor (2.16 g, 10.0 mmol, 1.0 e.q) in hot ethanol (40 ml). The mixture was heated (353 K with continuous stirring for 15 min and later allowed to cool to room temperature and stand for about 20 min., until a precipitate formed, which was then collected by filteration and dried over silica gel. The crude solid was purified by recrystallization from ethanol solution to give a yellow solid (3.39 g, yield: 80%). m.p 499–500 K. Elemental analysis calculated for C_16_H_11_BrFN_3_OS_2_: C, 45.29; H, 2.61; N, 9.90%. Found: C, 45.60; H, 2.31; N, 9.74%. FT–IR (KBr, *ν*, cm^−1^): 3157 (NH), 1694 (C=O); 1613 (C=N); 1068 (C=S); 1144 (N—N); ^1^H NMR (400 MHz, *d_6_*-DMSO) δ: (ppm): 4.56 (*s*, 2H), 6.90 (*d*, J = 8.2 Hz, 1H) 7.18–7.26 (*m*, 2H), 7.35–7.41 (*m*, 1H), 7.54–7.59 (*m*, 2H), 7.64 (*d*, *J* = 2.0 Hz, 1H), 11.48 (*s*, 1H), 13.89 (*s*, 1H). Crystals suitable for X-ray diffraction were grown by slow evaporation of an ethano­lic solution at room temperature.

## 6. Refinement

Crystal data, data collection, and structure refinement details are summarized in Table 3 ▸. The N-bound H atoms in **1** were located in a difference map and refined isotropically without restraint. The C-bound H atoms, and N-bond H atoms in **2**, were located geometrically (phenyl C—H = 0.95 Å, amide/thio­amide N—H = 0.88 Å, methyl­ene C—H = 0.99 Å, methyl C—H = 0.98 Å) and refined as riding atoms. The constraint *U*_iso_(H) = 1.2 *U*_eq_(carrier) or 1.5 *U*_eq_(methyl C) was applied in all cases. Non-merohedral twinning in the structure of **2** with the twin matrix [–1.000 0.000 0.000 / 0.000 1.001 −0.005 / 0.000 0.333 −1.001] was processed using the TwinRotMat routine in *PLATON* (Spek, 2009 ▸) and refined as a two component twin with component 2 rotated by 0.26° around [061] (reciprocal) or [010] (direct), with a refined twin fraction of 0.417 (6). This structure also showed disorder in its *o*-fluoro­benzyl group, with a 180° flip and a small (∼8°) twist around the S11—C11 bond. The aromatic ring and fluoro subsitutent was modelled in two parts with geometric and displacement-factor restraints retained on both major and minor parts in a 0.52 (3):0.48 (3) ratio.

## Supplementary Material

Crystal structure: contains datablock(s) 1, 2, general. DOI: 10.1107/S2056989025011028/hb8175sup1.cif

Structure factors: contains datablock(s) 1. DOI: 10.1107/S2056989025011028/hb81751sup2.hkl

Structure factors: contains datablock(s) 2. DOI: 10.1107/S2056989025011028/hb81752sup3.hkl

Supporting information file. DOI: 10.1107/S2056989025011028/hb81751sup4.cml

Supporting information file. DOI: 10.1107/S2056989025011028/hb81752sup5.cml

CCDC references: 2513536, 2513535

Additional supporting information:  crystallographic information; 3D view; checkCIF report

## Figures and Tables

**Table 1:** Hydrogen-bond geometry (Å, °) for **1**[Chem scheme1]

*D*—H⋯*A*	*D*—H	H⋯*A*	*D*⋯*A*	*D*—H⋯*A*
N1—H1⋯O21	0.78 (3)	2.05 (3)	2.820 (3)	174 (3)
N4—H4⋯O2	0.88 (3)	1.99 (3)	2.696 (3)	136 (3)
C7—H7⋯S10^i^	0.95	2.94	3.860 (3)	163
C21—H21*C*⋯O2^ii^	0.98	2.53	3.305 (3)	136
C22—H22*C*⋯O21^iii^	0.98	2.48	3.457 (3)	172

Symmetry codes: (i) 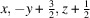; (ii) 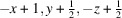; (iii) 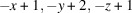.

**Table 2:** Hydrogen-bond geometry (Å, °) for (2)

*D*—H⋯*A*	*D*—H	H⋯*A*	*D*⋯*A*	*D*—H⋯*A*
N1—H1⋯O2^i^	0.88	1.96	2.821 (15)	165
N4—H4⋯O2	0.88	2.09	2.777 (16)	134
C11—H11*D*⋯S10	0.99	2.56	3.221 (14)	124

Symmetry code: (i) 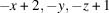.

**Table 3:** Experimental details

	**1**	**2**
Crystal data		
Chemical formula	C_16_H_12_FN_3_OS_2_·C_2_H_6_OS	C_16_H_11_BrFN_3_OS_2_
*M*_r_	423.53	424.31
Crystal system, space group	Monoclinic, *P*2_1_/*c*	Triclinic, *P* 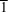
Temperature (K)	100	100
*a*, *b*, *c* (Å)	21.6312 (10), 4.6850 (2), 18.9579 (8)	6.6979 (7), 8.0075 (9), 15.880 (2)
α, β, γ (°)	90, 91.519 (4), 90	84.917 (10), 80.544 (10), 89.948 (9)
*V* (Å^3^)	1920.58 (14)	836.73 (17)
*Z*	4	2
Radiation type	Mo *K*α	Cu *K*α
μ (mm^−1^)	0.41	5.86
Crystal size (mm)	0.27 × 0.02 × 0.01	0.1 × 0.01 × 0.01

Data collection		
Diffractometer	Rigaku XtaLAB P200K	Rigaku XtaLAB P200K
Absorption correction	Multi-scan (*CrysAlis PRO*; Rigaku OD, 2024 ▸)	Multi-scan (*CrysAlis PRO*; Rigaku OD, 2024 ▸)
*T*_min_, *T*_max_	0.588, 1.000	0.695, 1.000
No. of measured, independent and observed [*I* > 2σ(*I*)] reflections	23801, 4647, 3366	12408, 3262, 1900
*R*_int_	0.069	0.139

Refinement		
*R*[*F*^2^ > 2σ(*F*^2^)], *wR*(*F*^2^), *S*	0.055, 0.114, 1.02	0.132, 0.319, 1.16
No. of reflections	4647	3262
No. of parameters	254	282
No. of restraints	0	130
H-atom treatment	H atoms treated by a mixture of independent and constrained refinement	H-atom parameters constrained
Δρ_max_, Δρ_min_ (e Å^−3^)	0.51, −0.32	1.39, −1.23

Computer programs: *CrysAlis PRO* (Rigaku OD, 2023 ▸, 2024 ▸), *SHELXT2018/2* (Sheldrick, 2015*a* ▸), *SHELXL2019/3* (Sheldrick, 2015*b* ▸), *Mercury* (Macrae *et al.*, 2020 ▸), *enCIFer* (Allen *et al.*, 2004 ▸) *publCIF* (Westrip, 2010 ▸) and *OLEX2* (Dolomanov *et al.*, 2009 ▸).

## supplementary crystallographic information

### 2-Fluorobenzyl (Z)-2-(2-oxoindolin-3-ylidene)hydrazine-1-carbodithioate dimethyl sulfoxide monololvate (1) Crystal data

**Table:**

C_16_H_12_FN_3_OS_2_·C_2_H_6_OS	*F*(000) = 880
*M**_r_* = 423.53	*D*_x_ = 1.465 Mg m^−^^3^
Monoclinic, *P*2_1_/*c*	Mo *K*α radiation, λ = 0.71073 Å
*a* = 21.6312 (10) Å	Cell parameters from 5747 reflections
*b* = 4.6850 (2) Å	θ = 2.2–26.9°
*c* = 18.9579 (8) Å	µ = 0.41 mm^−^^1^
β = 91.519 (4)°	*T* = 100 K
*V* = 1920.58 (14) Å^3^	Needle, yellow
*Z* = 4	0.27 × 0.02 × 0.01 mm

### 2-Fluorobenzyl (Z)-2-(2-oxoindolin-3-ylidene)hydrazine-1-carbodithioate dimethyl sulfoxide monololvate (1) Data collection

**Table:**

Rigaku XtaLAB P200K diffractometer	4647 independent reflections
Radiation source: Rotating Anode, Rigaku FR-X	3366 reflections with *I* > 2σ(*I*)
Rigaku Osmic Confocal Optical System monochromator	*R*_int_ = 0.069
Detector resolution: 5.8140 pixels mm^-1^	θ_max_ = 29.4°, θ_min_ = 2.2°
shutterless scans	*h* = −29→27
Absorption correction: multi-scan (CrysAlisPro; Rigaku OD, 2024)	*k* = −6→6
*T*_min_ = 0.588, *T*_max_ = 1.000	*l* = −25→24
23801 measured reflections	

### 2-Fluorobenzyl (Z)-2-(2-oxoindolin-3-ylidene)hydrazine-1-carbodithioate dimethyl sulfoxide monololvate (1) Refinement

**Table:**

Refinement on *F*^2^	Primary atom site location: dual
Least-squares matrix: full	Hydrogen site location: mixed
*R*[*F*^2^ > 2σ(*F*^2^)] = 0.055	H atoms treated by a mixture of independent and constrained refinement
*wR*(*F*^2^) = 0.114	*w* = 1/[σ^2^(*F*_o_^2^) + (0.0464*P*)^2^ + 1.708*P*] where *P* = (*F*_o_^2^ + 2*F*_c_^2^)/3
*S* = 1.02	(Δ/σ)_max_ = 0.001
4647 reflections	Δρ_max_ = 0.51 e Å^−^^3^
254 parameters	Δρ_min_ = −0.32 e Å^−^^3^
0 restraints	

### 2-Fluorobenzyl (Z)-2-(2-oxoindolin-3-ylidene)hydrazine-1-carbodithioate dimethyl sulfoxide monololvate (1) Special details

**Table:**

Geometry. All esds (except the esd in the dihedral angle between two l.s. planes) are estimated using the full covariance matrix. The cell esds are taken into account individually in the estimation of esds in distances, angles and torsion angles; correlations between esds in cell parameters are only used when they are defined by crystal symmetry. An approximate (isotropic) treatment of cell esds is used for estimating esds involving l.s. planes.
Refinement. Hydrogen atoms on N1 and N4 were located from the F_map_ and refined isotropically without restraint. DMSO solvate in the asymmetric unit is positioned outside the cell to clearly show discrete hydrogen bonding interaction between O21 and N1

### 2-Fluorobenzyl (Z)-2-(2-oxoindolin-3-ylidene)hydrazine-1-carbodithioate dimethyl sulfoxide monololvate (1) Fractional atomic coordinates and isotropic or equivalent isotropic displacement parameters (Å^2^)

**Table:**

	*x*	*y*	*z*	*U*_iso_*/*U*_eq_	
S10	0.71801 (3)	−0.17029 (15)	0.13746 (3)	0.02101 (17)	
S11	0.83457 (3)	0.09873 (15)	0.19938 (3)	0.02130 (17)	
F13	0.93202 (7)	0.2497 (4)	0.04837 (8)	0.0317 (4)	
O2	0.61299 (8)	0.3901 (4)	0.28556 (9)	0.0196 (4)	
N1	0.63062 (11)	0.7489 (5)	0.36760 (12)	0.0188 (5)	
H1	0.5968 (15)	0.793 (7)	0.3747 (17)	0.037 (10)*	
N3	0.75269 (9)	0.3900 (5)	0.28429 (10)	0.0176 (5)	
N4	0.72281 (10)	0.2053 (5)	0.23945 (11)	0.0184 (5)	
H4	0.6820 (16)	0.196 (7)	0.2376 (16)	0.040 (9)*	
C2	0.64701 (11)	0.5455 (5)	0.32149 (13)	0.0169 (5)	
C3	0.71713 (11)	0.5460 (5)	0.32265 (12)	0.0156 (5)	
C4	0.73668 (11)	0.7572 (5)	0.37423 (12)	0.0164 (5)	
C5	0.79386 (12)	0.8519 (6)	0.39943 (13)	0.0211 (6)	
H5	0.830912	0.776224	0.381188	0.025*	
C6	0.79602 (12)	1.0589 (6)	0.45168 (14)	0.0238 (6)	
H6	0.834959	1.123396	0.469705	0.029*	
C7	0.74203 (13)	1.1734 (6)	0.47810 (14)	0.0231 (6)	
H7	0.744531	1.313797	0.514229	0.028*	
C8	0.68421 (12)	1.0844 (6)	0.45213 (13)	0.0213 (6)	
H8	0.647223	1.164628	0.469411	0.026*	
C9	0.68241 (11)	0.8767 (5)	0.40063 (12)	0.0163 (5)	
C10	0.75457 (11)	0.0472 (5)	0.19318 (12)	0.0172 (5)	
C11	0.86058 (12)	−0.1306 (6)	0.12843 (14)	0.0239 (6)	
H11A	0.845435	−0.327976	0.135090	0.029*	
H11B	0.844624	−0.059551	0.082264	0.029*	
C12	0.93023 (12)	−0.1248 (6)	0.13096 (13)	0.0207 (6)	
C13	0.96358 (12)	0.0615 (6)	0.09076 (14)	0.0226 (6)	
C14	1.02731 (12)	0.0685 (6)	0.09125 (14)	0.0267 (6)	
H14	1.048630	0.199412	0.062354	0.032*	
C15	1.05949 (12)	−0.1200 (6)	0.13488 (15)	0.0271 (6)	
H15	1.103426	−0.119825	0.136062	0.032*	
C16	1.02768 (12)	−0.3084 (6)	0.17671 (15)	0.0264 (6)	
H16	1.049759	−0.437167	0.206774	0.032*	
C17	0.96361 (12)	−0.3096 (6)	0.17480 (14)	0.0237 (6)	
H17	0.942143	−0.438896	0.203984	0.028*	
S21	0.50131 (3)	1.27070 (14)	0.39185 (3)	0.01653 (16)	
O21	0.50964 (8)	0.9499 (4)	0.38887 (9)	0.0216 (4)	
C21	0.46100 (12)	1.3699 (6)	0.31267 (12)	0.0199 (6)	
H21A	0.488204	1.345701	0.272589	0.030*	
H21B	0.448170	1.570006	0.315740	0.030*	
H21C	0.424392	1.248576	0.306044	0.030*	
C22	0.43948 (11)	1.3253 (6)	0.45068 (13)	0.0226 (6)	
H22A	0.403487	1.213317	0.434743	0.034*	
H22B	0.428535	1.528252	0.451386	0.034*	
H22C	0.452432	1.264394	0.498271	0.034*	

### 2-Fluorobenzyl (Z)-2-(2-oxoindolin-3-ylidene)hydrazine-1-carbodithioate dimethyl sulfoxide monololvate (1) Atomic displacement parameters (Å^2^)

**Table:**

	*U*^11^	*U*^22^	*U*^33^	*U*^12^	*U*^13^	*U*^23^
S10	0.0165 (3)	0.0279 (4)	0.0185 (3)	−0.0028 (3)	−0.0018 (2)	−0.0033 (3)
S11	0.0125 (3)	0.0290 (4)	0.0224 (3)	−0.0004 (3)	0.0002 (2)	−0.0075 (3)
F13	0.0314 (10)	0.0376 (10)	0.0260 (9)	0.0043 (8)	−0.0014 (7)	0.0036 (8)
O2	0.0134 (9)	0.0255 (10)	0.0197 (9)	−0.0011 (8)	−0.0014 (7)	−0.0019 (8)
N1	0.0121 (11)	0.0216 (12)	0.0228 (12)	0.0015 (10)	0.0022 (9)	−0.0021 (10)
N3	0.0164 (11)	0.0197 (11)	0.0165 (10)	−0.0025 (9)	−0.0009 (8)	0.0010 (9)
N4	0.0126 (11)	0.0246 (13)	0.0178 (11)	−0.0019 (9)	−0.0012 (9)	−0.0017 (9)
C2	0.0139 (12)	0.0195 (14)	0.0173 (12)	0.0012 (11)	−0.0002 (10)	0.0039 (11)
C3	0.0125 (12)	0.0188 (13)	0.0157 (12)	0.0001 (10)	0.0007 (9)	0.0020 (11)
C4	0.0181 (13)	0.0181 (13)	0.0131 (12)	0.0005 (11)	0.0009 (10)	0.0009 (10)
C5	0.0172 (13)	0.0244 (15)	0.0217 (13)	0.0011 (11)	−0.0003 (10)	0.0020 (12)
C6	0.0219 (14)	0.0271 (16)	0.0221 (14)	−0.0054 (12)	−0.0038 (11)	−0.0006 (12)
C7	0.0279 (15)	0.0236 (15)	0.0177 (13)	−0.0037 (12)	0.0001 (11)	−0.0019 (11)
C8	0.0213 (14)	0.0232 (15)	0.0195 (13)	0.0001 (11)	0.0054 (10)	0.0015 (12)
C9	0.0151 (12)	0.0163 (13)	0.0176 (13)	−0.0004 (10)	0.0016 (9)	0.0032 (11)
C10	0.0156 (13)	0.0222 (14)	0.0139 (12)	−0.0002 (11)	0.0006 (10)	0.0040 (11)
C11	0.0178 (14)	0.0314 (16)	0.0224 (14)	0.0011 (12)	−0.0008 (10)	−0.0078 (12)
C12	0.0166 (13)	0.0264 (15)	0.0189 (13)	0.0041 (11)	−0.0007 (10)	−0.0087 (12)
C13	0.0221 (14)	0.0247 (15)	0.0209 (14)	0.0041 (12)	−0.0013 (11)	−0.0049 (12)
C14	0.0229 (15)	0.0321 (17)	0.0253 (15)	−0.0045 (13)	0.0061 (11)	−0.0058 (13)
C15	0.0157 (13)	0.0325 (17)	0.0331 (16)	0.0014 (12)	0.0007 (11)	−0.0099 (14)
C16	0.0194 (14)	0.0293 (16)	0.0302 (15)	0.0053 (12)	−0.0054 (11)	−0.0044 (13)
C17	0.0220 (14)	0.0262 (15)	0.0229 (14)	−0.0018 (12)	0.0004 (11)	−0.0039 (12)
S21	0.0130 (3)	0.0207 (4)	0.0158 (3)	0.0007 (3)	−0.0007 (2)	−0.0009 (3)
O21	0.0183 (10)	0.0216 (10)	0.0248 (10)	0.0020 (8)	0.0005 (7)	−0.0001 (8)
C21	0.0182 (13)	0.0252 (15)	0.0163 (13)	0.0024 (11)	−0.0003 (10)	−0.0002 (11)
C22	0.0178 (13)	0.0327 (16)	0.0175 (13)	0.0037 (12)	0.0026 (10)	0.0004 (12)

### 2-Fluorobenzyl (Z)-2-(2-oxoindolin-3-ylidene)hydrazine-1-carbodithioate dimethyl sulfoxide monololvate (1) Geometric parameters (Å, º)

**Table:**

S10—C10	1.654 (3)	C8—C9	1.378 (4)
S11—C10	1.748 (2)	C11—H11A	0.9900
S11—C11	1.822 (3)	C11—H11B	0.9900
F13—C13	1.364 (3)	C11—C12	1.506 (4)
O2—C2	1.228 (3)	C12—C13	1.376 (4)
N1—H1	0.78 (3)	C12—C17	1.389 (4)
N1—C2	1.347 (3)	C13—C14	1.379 (4)
N1—C9	1.403 (3)	C14—H14	0.9500
N3—N4	1.364 (3)	C14—C15	1.385 (4)
N3—C3	1.298 (3)	C15—H15	0.9500
N4—H4	0.88 (3)	C15—C16	1.382 (4)
N4—C10	1.350 (3)	C16—H16	0.9500
C2—C3	1.517 (3)	C16—C17	1.385 (4)
C3—C4	1.446 (3)	C17—H17	0.9500
C4—C5	1.387 (3)	S21—O21	1.5150 (19)
C4—C9	1.405 (3)	S21—C21	1.778 (2)
C5—H5	0.9500	S21—C22	1.783 (2)
C5—C6	1.386 (4)	C21—H21A	0.9800
C6—H6	0.9500	C21—H21B	0.9800
C6—C7	1.391 (4)	C21—H21C	0.9800
C7—H7	0.9500	C22—H22A	0.9800
C7—C8	1.396 (4)	C22—H22B	0.9800
C8—H8	0.9500	C22—H22C	0.9800
			
C10—S11—C11	101.15 (12)	H11A—C11—H11B	108.6
C2—N1—H1	125 (2)	C12—C11—S11	107.09 (18)
C2—N1—C9	111.8 (2)	C12—C11—H11A	110.3
C9—N1—H1	124 (2)	C12—C11—H11B	110.3
C3—N3—N4	115.4 (2)	C13—C12—C11	122.2 (2)
N3—N4—H4	121 (2)	C13—C12—C17	117.1 (2)
C10—N4—N3	120.8 (2)	C17—C12—C11	120.8 (3)
C10—N4—H4	118 (2)	F13—C13—C12	118.4 (2)
O2—C2—N1	128.0 (2)	F13—C13—C14	118.3 (2)
O2—C2—C3	126.4 (2)	C12—C13—C14	123.4 (3)
N1—C2—C3	105.6 (2)	C13—C14—H14	120.8
N3—C3—C2	126.8 (2)	C13—C14—C15	118.4 (3)
N3—C3—C4	126.7 (2)	C15—C14—H14	120.8
C4—C3—C2	106.6 (2)	C14—C15—H15	120.0
C5—C4—C3	133.9 (2)	C16—C15—C14	120.0 (3)
C5—C4—C9	119.7 (2)	C16—C15—H15	120.0
C9—C4—C3	106.3 (2)	C15—C16—H16	120.0
C4—C5—H5	120.6	C15—C16—C17	120.0 (3)
C6—C5—C4	118.9 (2)	C17—C16—H16	120.0
C6—C5—H5	120.6	C12—C17—H17	119.4
C5—C6—H6	119.5	C16—C17—C12	121.1 (3)
C5—C6—C7	121.0 (2)	C16—C17—H17	119.4
C7—C6—H6	119.5	O21—S21—C21	106.50 (12)
C6—C7—H7	119.6	O21—S21—C22	104.93 (12)
C6—C7—C8	120.7 (2)	C21—S21—C22	97.55 (12)
C8—C7—H7	119.6	S21—C21—H21A	109.5
C7—C8—H8	121.0	S21—C21—H21B	109.5
C9—C8—C7	118.0 (2)	S21—C21—H21C	109.5
C9—C8—H8	121.0	H21A—C21—H21B	109.5
N1—C9—C4	109.6 (2)	H21A—C21—H21C	109.5
C8—C9—N1	128.7 (2)	H21B—C21—H21C	109.5
C8—C9—C4	121.7 (2)	S21—C22—H22A	109.5
S10—C10—S11	125.71 (15)	S21—C22—H22B	109.5
N4—C10—S10	120.67 (19)	S21—C22—H22C	109.5
N4—C10—S11	113.62 (18)	H22A—C22—H22B	109.5
S11—C11—H11A	110.3	H22A—C22—H22C	109.5
S11—C11—H11B	110.3	H22B—C22—H22C	109.5
			
S11—C11—C12—C13	−93.9 (3)	C5—C4—C9—N1	179.9 (2)
S11—C11—C12—C17	85.9 (3)	C5—C4—C9—C8	−1.0 (4)
F13—C13—C14—C15	179.2 (2)	C5—C6—C7—C8	−0.6 (4)
O2—C2—C3—N3	2.5 (4)	C6—C7—C8—C9	1.2 (4)
O2—C2—C3—C4	−178.2 (2)	C7—C8—C9—N1	178.5 (2)
N1—C2—C3—N3	−177.4 (2)	C7—C8—C9—C4	−0.4 (4)
N1—C2—C3—C4	1.8 (3)	C9—N1—C2—O2	178.1 (2)
N3—N4—C10—S10	−178.38 (18)	C9—N1—C2—C3	−1.9 (3)
N3—N4—C10—S11	1.7 (3)	C9—C4—C5—C6	1.6 (4)
N3—C3—C4—C5	−1.8 (5)	C10—S11—C11—C12	−175.81 (19)
N3—C3—C4—C9	178.2 (2)	C11—S11—C10—S10	2.4 (2)
N4—N3—C3—C2	−1.0 (3)	C11—S11—C10—N4	−177.65 (19)
N4—N3—C3—C4	179.8 (2)	C11—C12—C13—F13	1.2 (4)
C2—N1—C9—C4	1.3 (3)	C11—C12—C13—C14	−179.0 (2)
C2—N1—C9—C8	−177.6 (2)	C11—C12—C17—C16	179.1 (2)
C2—C3—C4—C5	179.0 (3)	C12—C13—C14—C15	−0.6 (4)
C2—C3—C4—C9	−1.1 (3)	C13—C12—C17—C16	−1.1 (4)
C3—N3—N4—C10	175.0 (2)	C13—C14—C15—C16	−0.2 (4)
C3—C4—C5—C6	−178.5 (3)	C14—C15—C16—C17	0.3 (4)
C3—C4—C9—N1	−0.1 (3)	C15—C16—C17—C12	0.4 (4)
C3—C4—C9—C8	179.0 (2)	C17—C12—C13—F13	−178.6 (2)
C4—C5—C6—C7	−0.8 (4)	C17—C12—C13—C14	1.2 (4)

### 2-Fluorobenzyl (Z)-2-(2-oxoindolin-3-ylidene)hydrazine-1-carbodithioate dimethyl sulfoxide monololvate (1) Hydrogen-bond geometry (Å, º)

**Table:**

*D*—H···*A*	*D*—H	H···*A*	*D*···*A*	*D*—H···*A*
N1—H1···O21	0.78 (3)	2.05 (3)	2.820 (3)	174 (3)
N4—H4···O2	0.88 (3)	1.99 (3)	2.696 (3)	136 (3)
C7—H7···S10^i^	0.95	2.94	3.860 (3)	163
C21—H21*C*···O2^ii^	0.98	2.53	3.305 (3)	136
C22—H22*C*···O21^iii^	0.98	2.48	3.457 (3)	172

Symmetry codes: (i) *x*, −*y*+3/2, *z*+1/2; (ii) −*x*+1, *y*+1/2, −*z*+1/2; (iii) −*x*+1, −*y*+2, −*z*+1.

### 2-Fluorobenzyl (Z)-2-(5-bromo-2-oxoindolin-3-ylidene)hydrazine-1-carbodithioate (2) Crystal data

**Table:**

C_16_H_11_BrFN_3_OS_2_	*Z* = 2
*M**_r_* = 424.31	*F*(000) = 424
Triclinic, *P*1	*D*_x_ = 1.684 Mg m^−^^3^
*a* = 6.6979 (7) Å	Cu *K*α radiation, λ = 1.54184 Å
*b* = 8.0075 (9) Å	Cell parameters from 1932 reflections
*c* = 15.880 (2) Å	θ = 2.8–60.0°
α = 84.917 (10)°	µ = 5.86 mm^−^^1^
β = 80.544 (10)°	*T* = 100 K
γ = 89.948 (9)°	Needle, orange
*V* = 836.73 (17) Å^3^	0.1 × 0.01 × 0.01 mm

### 2-Fluorobenzyl (Z)-2-(5-bromo-2-oxoindolin-3-ylidene)hydrazine-1-carbodithioate (2) Data collection

**Table:**

Rigaku XtaLAB P200K diffractometer	3262 independent reflections
Radiation source: Rotating Anode, Rigaku MM-007HF	1900 reflections with *I* > 2σ(*I*)
Rigaku Osmic Confocal Optical System monochromator	*R*_int_ = 0.139
Detector resolution: 5.8140 pixels mm^-1^	θ_max_ = 75.8°, θ_min_ = 2.8°
shutterless scans	*h* = −8→8
Absorption correction: multi-scan (CrysAlisPro; Rigaku OD, 2024)	*k* = −9→10
*T*_min_ = 0.695, *T*_max_ = 1.000	*l* = −19→19
12408 measured reflections	

### 2-Fluorobenzyl (Z)-2-(5-bromo-2-oxoindolin-3-ylidene)hydrazine-1-carbodithioate (2) Refinement

**Table:**

Refinement on *F*^2^	Primary atom site location: dual
Least-squares matrix: full	Hydrogen site location: inferred from neighbouring sites
*R*[*F*^2^ > 2σ(*F*^2^)] = 0.132	H-atom parameters constrained
*wR*(*F*^2^) = 0.319	*w* = 1/[σ^2^(*F*_o_^2^) + 14.5238*P*] where *P* = (*F*_o_^2^ + 2*F*_c_^2^)/3
*S* = 1.16	(Δ/σ)_max_ < 0.001
3262 reflections	Δρ_max_ = 1.39 e Å^−^^3^
282 parameters	Δρ_min_ = −1.23 e Å^−^^3^
130 restraints	

### 2-Fluorobenzyl (Z)-2-(5-bromo-2-oxoindolin-3-ylidene)hydrazine-1-carbodithioate (2) Special details

**Table:**

Geometry. All esds (except the esd in the dihedral angle between two l.s. planes) are estimated using the full covariance matrix. The cell esds are taken into account individually in the estimation of esds in distances, angles and torsion angles; correlations between esds in cell parameters are only used when they are defined by crystal symmetry. An approximate (isotropic) treatment of cell esds is used for estimating esds involving l.s. planes.
Refinement. Refined as a 2-component twin at [-1 0 0 0 1.001 -0.005 0 0.333 -1.001] with HKLF5 generated by TWINROTMAT running in PLATON. Ortho-fluorobenzyl group was disordered with fluoro group fliped 180°. fluoro- phenyl ring was split in two parts and refined with C—F and aromatic C—C distances restrained, C12A/B thermal parameters linked with SIMU, and general thermal restraints on both parts.

### 2-Fluorobenzyl (Z)-2-(5-bromo-2-oxoindolin-3-ylidene)hydrazine-1-carbodithioate (2) Fractional atomic coordinates and isotropic or equivalent isotropic displacement parameters (Å^2^)

**Table:**

	*x*	*y*	*z*	*U*_iso_*/*U*_eq_	Occ. (<1)
Br6	0.0746 (3)	0.4658 (3)	0.34527 (14)	0.0526 (6)	
S10	0.5773 (6)	0.2120 (6)	0.8720 (3)	0.0532 (12)	
S11	0.2188 (6)	0.3262 (6)	0.7844 (3)	0.0493 (12)	
F13A	−0.190 (3)	0.587 (3)	0.8641 (17)	0.063 (8)	0.48 (3)
F13B	0.423 (3)	0.628 (3)	0.9211 (15)	0.066 (7)	0.52 (3)
O2	0.8662 (15)	0.0741 (14)	0.5997 (7)	0.042 (3)	
N1	0.7918 (17)	0.1135 (16)	0.4609 (9)	0.038 (3)	
H1	0.897901	0.066037	0.432771	0.046*	
N3	0.4653 (18)	0.2489 (16)	0.6398 (9)	0.040 (3)	
N4	0.5546 (17)	0.2100 (17)	0.7094 (8)	0.040 (3)	
H4	0.672455	0.159957	0.703457	0.048*	
C2	0.759 (2)	0.124 (2)	0.5459 (13)	0.044 (4)	
C3	0.560 (2)	0.2145 (19)	0.5670 (11)	0.038 (4)	
C4	0.491 (2)	0.256 (2)	0.4859 (11)	0.040 (4)	
C5	0.319 (2)	0.331 (2)	0.4627 (12)	0.044 (4)	
H5	0.214241	0.367252	0.504814	0.053*	
C6	0.303 (2)	0.350 (2)	0.3780 (14)	0.052 (5)	
C7	0.444 (2)	0.287 (2)	0.3158 (11)	0.042 (4)	
H7	0.425799	0.299914	0.257538	0.051*	
C8	0.614 (2)	0.204 (2)	0.3380 (12)	0.044 (4)	
H8	0.711650	0.159053	0.295954	0.053*	
C9	0.633 (2)	0.189 (2)	0.4224 (11)	0.043 (4)	
C10	0.465 (2)	0.247 (2)	0.7876 (13)	0.050 (5)	
C11	0.134 (2)	0.3751 (17)	0.8932 (11)	0.044 (4)	
H11A	−0.002943	0.326768	0.913682	0.052*	0.48 (3)
H11B	0.226375	0.323685	0.930561	0.052*	0.48 (3)
H11C	0.006433	0.313510	0.916922	0.052*	0.52 (3)
H11D	0.237024	0.339671	0.929080	0.052*	0.52 (3)
C12A	0.129 (7)	0.563 (3)	0.899 (7)	0.041 (9)	0.48 (3)
C12B	0.101 (7)	0.562 (3)	0.895 (7)	0.045 (9)	0.52 (3)
C13A	−0.028 (4)	0.667 (3)	0.881 (3)	0.048 (11)	0.48 (3)
C13B	0.242 (4)	0.681 (4)	0.905 (3)	0.044 (9)	0.52 (3)
C14A	−0.027 (6)	0.838 (4)	0.887 (3)	0.051 (10)	0.48 (3)
H14A	−0.141486	0.903603	0.878526	0.061*	0.48 (3)
C14B	0.201 (5)	0.849 (4)	0.913 (3)	0.053 (8)	0.52 (3)
H14B	0.298101	0.922374	0.927666	0.064*	0.52 (3)
C15A	0.145 (5)	0.910 (4)	0.906 (3)	0.055 (11)	0.48 (3)
H15A	0.156351	1.028798	0.905431	0.066*	0.48 (3)
C15B	0.015 (5)	0.905 (4)	0.898 (3)	0.051 (8)	0.52 (3)
H15B	−0.013716	1.020749	0.900113	0.061*	0.52 (3)
C16A	0.302 (6)	0.808 (4)	0.925 (3)	0.061 (11)	0.48 (3)
H16A	0.417329	0.858294	0.940689	0.073*	0.48 (3)
C16B	−0.131 (5)	0.798 (3)	0.880 (2)	0.049 (8)	0.52 (3)
H16B	−0.255576	0.837253	0.866029	0.059*	0.52 (3)
C17A	0.296 (6)	0.638 (4)	0.922 (3)	0.046 (9)	0.48 (3)
H17A	0.406788	0.571999	0.934537	0.056*	0.48 (3)
C17B	−0.085 (6)	0.631 (4)	0.884 (3)	0.044 (8)	0.52 (3)
H17B	−0.189861	0.554327	0.879354	0.053*	0.52 (3)

### 2-Fluorobenzyl (Z)-2-(5-bromo-2-oxoindolin-3-ylidene)hydrazine-1-carbodithioate (2) Atomic displacement parameters (Å^2^)

**Table:**

	*U*^11^	*U*^22^	*U*^33^	*U*^12^	*U*^13^	*U*^23^
Br6	0.0318 (8)	0.0514 (12)	0.0740 (14)	0.0068 (8)	−0.0112 (8)	0.0025 (10)
S10	0.049 (2)	0.046 (3)	0.066 (3)	0.007 (2)	−0.016 (2)	−0.006 (2)
S11	0.046 (2)	0.042 (3)	0.059 (3)	0.0194 (19)	−0.006 (2)	−0.005 (2)
F13A	0.041 (11)	0.043 (12)	0.11 (2)	−0.002 (8)	−0.026 (11)	0.003 (12)
F13B	0.053 (11)	0.070 (14)	0.084 (16)	0.013 (9)	−0.026 (11)	−0.037 (12)
O2	0.035 (5)	0.039 (7)	0.053 (7)	0.012 (5)	−0.004 (5)	−0.012 (6)
N1	0.029 (6)	0.027 (7)	0.057 (9)	0.003 (5)	−0.003 (6)	−0.006 (6)
N3	0.038 (7)	0.025 (7)	0.056 (9)	0.003 (6)	−0.004 (6)	−0.008 (7)
N4	0.030 (6)	0.040 (8)	0.047 (8)	0.009 (6)	0.001 (6)	−0.009 (7)
C2	0.023 (7)	0.029 (9)	0.077 (14)	0.000 (6)	0.001 (8)	−0.002 (9)
C3	0.029 (7)	0.026 (8)	0.061 (11)	0.006 (6)	−0.002 (7)	−0.020 (8)
C4	0.031 (7)	0.036 (9)	0.050 (10)	−0.006 (7)	−0.002 (7)	0.003 (8)
C5	0.038 (8)	0.034 (9)	0.058 (11)	0.008 (7)	−0.001 (8)	−0.004 (8)
C6	0.019 (7)	0.037 (10)	0.096 (15)	0.013 (6)	−0.007 (8)	0.005 (10)
C7	0.031 (8)	0.040 (10)	0.056 (11)	−0.013 (7)	−0.014 (7)	0.008 (8)
C8	0.034 (8)	0.034 (9)	0.063 (12)	0.008 (7)	−0.004 (8)	−0.011 (9)
C9	0.032 (8)	0.045 (10)	0.053 (11)	−0.002 (7)	−0.008 (7)	−0.002 (9)
C10	0.039 (9)	0.025 (9)	0.081 (14)	0.010 (7)	−0.004 (9)	0.004 (9)
C11	0.048 (9)	0.031 (9)	0.049 (10)	0.001 (7)	0.002 (8)	−0.008 (8)
C12A	0.032 (14)	0.040 (14)	0.05 (2)	0.009 (10)	0.004 (14)	−0.001 (15)
C12B	0.039 (12)	0.042 (12)	0.05 (2)	0.002 (9)	−0.004 (13)	−0.003 (14)
C13A	0.036 (14)	0.032 (12)	0.08 (3)	0.005 (9)	−0.009 (15)	−0.007 (14)
C13B	0.039 (11)	0.044 (11)	0.05 (2)	−0.003 (8)	−0.002 (11)	0.004 (13)
C14A	0.051 (18)	0.034 (12)	0.07 (3)	0.005 (10)	−0.014 (18)	−0.008 (15)
C14B	0.053 (10)	0.049 (10)	0.059 (13)	−0.001 (7)	−0.012 (8)	−0.008 (9)
C15A	0.058 (19)	0.029 (14)	0.08 (3)	−0.003 (11)	−0.022 (17)	0.003 (17)
C15B	0.052 (10)	0.045 (10)	0.053 (12)	0.003 (7)	−0.006 (8)	−0.004 (9)
C16A	0.060 (18)	0.041 (14)	0.08 (3)	−0.002 (13)	−0.026 (19)	0.009 (16)
C16B	0.042 (13)	0.044 (11)	0.06 (2)	0.006 (9)	0.001 (14)	−0.011 (12)
C17A	0.049 (18)	0.038 (13)	0.05 (2)	−0.003 (12)	−0.013 (18)	0.020 (15)
C17B	0.042 (10)	0.043 (10)	0.049 (12)	0.005 (7)	−0.008 (9)	−0.008 (9)

### 2-Fluorobenzyl (Z)-2-(5-bromo-2-oxoindolin-3-ylidene)hydrazine-1-carbodithioate (2) Geometric parameters (Å, º)

**Table:**

Br6—C6	1.910 (14)	C11—H11B	0.9900
S10—C10	1.644 (19)	C11—H11C	0.9900
S11—C10	1.774 (15)	C11—H11D	0.9900
S11—C11	1.804 (16)	C11—C12A	1.519 (19)
F13A—C13A	1.342 (19)	C11—C12B	1.516 (18)
F13B—C13B	1.339 (18)	C12A—C13A	1.390 (19)
O2—C2	1.246 (19)	C12A—C17A	1.38 (2)
N1—H1	0.8800	C12B—C13B	1.383 (19)
N1—C2	1.34 (2)	C12B—C17B	1.385 (19)
N1—C9	1.421 (19)	C13A—C14A	1.382 (19)
N3—N4	1.353 (18)	C13B—C14B	1.381 (19)
N3—C3	1.28 (2)	C14A—H14A	0.9500
N4—H4	0.8800	C14A—C15A	1.38 (2)
N4—C10	1.34 (2)	C14B—H14B	0.9500
C2—C3	1.517 (19)	C14B—C15B	1.372 (19)
C3—C4	1.45 (2)	C15A—H15A	0.9500
C4—C5	1.39 (2)	C15A—C16A	1.386 (19)
C4—C9	1.41 (2)	C15B—H15B	0.9500
C5—H5	0.9500	C15B—C16B	1.38 (2)
C5—C6	1.36 (3)	C16A—H16A	0.9500
C6—C7	1.38 (2)	C16A—C17A	1.369 (19)
C7—H7	0.9500	C16B—H16B	0.9500
C7—C8	1.39 (2)	C16B—C17B	1.370 (19)
C8—H8	0.9500	C17A—H17A	0.9500
C8—C9	1.36 (2)	C17B—H17B	0.9500
C11—H11A	0.9900		
			
C10—S11—C11	103.1 (9)	C12A—C11—H11A	109.4
C2—N1—H1	124.5	C12A—C11—H11B	109.4
C2—N1—C9	111.0 (13)	C12B—C11—S11	110 (4)
C9—N1—H1	124.5	C12B—C11—H11C	109.7
C3—N3—N4	117.9 (13)	C12B—C11—H11D	109.7
N3—N4—H4	119.6	C13A—C12A—C11	125 (3)
C10—N4—N3	120.7 (12)	C17A—C12A—C11	118 (2)
C10—N4—H4	119.6	C17A—C12A—C13A	117 (2)
O2—C2—N1	128.7 (14)	C13B—C12B—C11	126 (3)
O2—C2—C3	124.4 (16)	C13B—C12B—C17B	113 (2)
N1—C2—C3	106.9 (14)	C17B—C12B—C11	121 (2)
N3—C3—C2	129.1 (16)	F13A—C13A—C12A	115 (2)
N3—C3—C4	124.8 (13)	F13A—C13A—C14A	121 (3)
C4—C3—C2	106.1 (14)	C14A—C13A—C12A	123 (2)
C5—C4—C3	134.1 (15)	F13B—C13B—C12B	118 (3)
C5—C4—C9	119.0 (16)	F13B—C13B—C14B	117 (3)
C9—C4—C3	106.7 (13)	C14B—C13B—C12B	124 (3)
C4—C5—H5	120.9	C13A—C14A—H14A	121.1
C6—C5—C4	118.2 (15)	C15A—C14A—C13A	118 (3)
C6—C5—H5	120.9	C15A—C14A—H14A	121.1
C5—C6—Br6	118.3 (13)	C13B—C14B—H14B	121.1
C5—C6—C7	122.4 (14)	C15B—C14B—C13B	118 (3)
C7—C6—Br6	119.3 (15)	C15B—C14B—H14B	121.1
C6—C7—H7	119.8	C14A—C15A—H15A	120.4
C6—C7—C8	120.3 (17)	C14A—C15A—C16A	119 (3)
C8—C7—H7	119.8	C16A—C15A—H15A	120.4
C7—C8—H8	121.3	C14B—C15B—H15B	119.1
C9—C8—C7	117.4 (16)	C14B—C15B—C16B	122 (3)
C9—C8—H8	121.3	C16B—C15B—H15B	119.1
C4—C9—N1	109.3 (14)	C15A—C16A—H16A	118.9
C8—C9—N1	128.2 (15)	C17A—C16A—C15A	122 (4)
C8—C9—C4	122.4 (15)	C17A—C16A—H16A	118.9
S10—C10—S11	127.5 (12)	C15B—C16B—H16B	121.9
N4—C10—S10	121.5 (11)	C17B—C16B—C15B	116 (3)
N4—C10—S11	110.9 (13)	C17B—C16B—H16B	121.9
S11—C11—H11A	109.4	C12A—C17A—H17A	120.1
S11—C11—H11B	109.4	C16A—C17A—C12A	120 (3)
S11—C11—H11C	109.7	C16A—C17A—H17A	120.1
S11—C11—H11D	109.7	C12B—C17B—H17B	116.9
H11A—C11—H11B	108.0	C16B—C17B—C12B	126 (3)
H11C—C11—H11D	108.2	C16B—C17B—H17B	116.9
C12A—C11—S11	111 (4)		
			
Br6—C6—C7—C8	179.3 (12)	C6—C7—C8—C9	0 (2)
S11—C11—C12A—C13A	82 (9)	C7—C8—C9—N1	−178.7 (15)
S11—C11—C12A—C17A	−95 (7)	C7—C8—C9—C4	−1 (2)
S11—C11—C12B—C13B	−91 (9)	C9—N1—C2—O2	−179.0 (16)
S11—C11—C12B—C17B	88 (8)	C9—N1—C2—C3	0.1 (17)
F13A—C13A—C14A—C15A	180 (4)	C9—C4—C5—C6	−6 (2)
F13B—C13B—C14B—C15B	−179 (4)	C10—S11—C11—C12A	107.9 (17)
O2—C2—C3—N3	−3 (3)	C10—S11—C11—C12B	116.4 (16)
O2—C2—C3—C4	177.5 (15)	C11—S11—C10—S10	5.0 (14)
N1—C2—C3—N3	178.0 (15)	C11—S11—C10—N4	−177.6 (11)
N1—C2—C3—C4	−1.6 (17)	C11—C12A—C13A—F13A	6 (11)
N3—N4—C10—S10	−176.1 (12)	C11—C12A—C13A—C14A	−179 (7)
N3—N4—C10—S11	6.3 (19)	C11—C12A—C17A—C16A	177 (6)
N3—C3—C4—C5	−3 (3)	C11—C12B—C13B—F13B	−5 (11)
N3—C3—C4—C9	−177.2 (16)	C11—C12B—C13B—C14B	−175 (7)
N4—N3—C3—C2	4 (2)	C11—C12B—C17B—C16B	−176 (6)
N4—N3—C3—C4	−176.4 (14)	C12A—C13A—C14A—C15A	5 (9)
C2—N1—C9—C4	1.4 (18)	C12B—C13B—C14B—C15B	−9 (8)
C2—N1—C9—C8	179.6 (17)	C13A—C12A—C17A—C16A	0 (11)
C2—C3—C4—C5	176.7 (18)	C13A—C14A—C15A—C16A	−5 (7)
C2—C3—C4—C9	2.4 (17)	C13B—C12B—C17B—C16B	2 (11)
C3—N3—N4—C10	178.2 (15)	C13B—C14B—C15B—C16B	3 (6)
C3—C4—C5—C6	−179.8 (17)	C14A—C15A—C16A—C17A	3 (7)
C3—C4—C9—N1	−2.4 (18)	C14B—C15B—C16B—C17B	5 (6)
C3—C4—C9—C8	179.3 (15)	C15A—C16A—C17A—C12A	−1 (8)
C4—C5—C6—Br6	−176.1 (12)	C15B—C16B—C17B—C12B	−8 (8)
C4—C5—C6—C7	5 (3)	C17A—C12A—C13A—F13A	−177 (6)
C5—C4—C9—N1	−177.7 (14)	C17A—C12A—C13A—C14A	−2 (11)
C5—C4—C9—C8	4 (3)	C17B—C12B—C13B—F13B	176 (6)
C5—C6—C7—C8	−2 (3)	C17B—C12B—C13B—C14B	6 (11)

### 2-Fluorobenzyl (Z)-2-(5-bromo-2-oxoindolin-3-ylidene)hydrazine-1-carbodithioate (2) Hydrogen-bond geometry (Å, º)

**Table:**

*D*—H···*A*	*D*—H	H···*A*	*D*···*A*	*D*—H···*A*
N1—H1···O2^i^	0.88	1.96	2.821 (15)	165
N4—H4···O2	0.88	2.09	2.777 (16)	134
C11—H11*D*···S10	0.99	2.56	3.221 (14)	124

Symmetry code: (i) −*x*+2, −*y*, −*z*+1.
